# Implementing Fabrication as a Pedagogical Tool in Vertebrate Anatomy Courses: Motivation, Inclusion, and Lessons

**DOI:** 10.1093/icb/icab147

**Published:** 2021-06-26

**Authors:** Katie Lynn Staab

**Affiliations:** Biology Department, McDaniel College, Westminster, MD 21157, USA

## Abstract

Increasing course structure by incorporating active learning and multimodal pedagogical strategies benefits all learners. Students of vertebrate anatomy can especially benefit from practicing fabrication, or “making”, incorporating skills such as 3D digital modeling, 3D printing, and using familiar low-tech materials to construct informed replicas of animal anatomy. Student perceptions of active learning projects are shaped by motivation theories such as the expectancy-value theory and self-directed learning, both of which are briefly reviewed here. This paper offers inspiration and resources to instructors for establishing a makerspace in an anatomy lab and leveraging community partners to stimulate students to construct their own versions of nature's designs. Learning science in informal environments and specifically in makerspaces has been shown to promote equity and increase motivation to study science. Examples here emphasize accessibility for diverse learners, including strategies for instructors to ensure ease of student access to 3D technology. Scaffolding formative assessments builds student confidence and expertise, further closing opportunity gaps. Two specific cases are detailed where fabrication and the use of 3D digital models are used to augment student learning of vertebrate anatomy at a small liberal arts college. In a semester-long research project in an introductory biomechanics course, students investigate, write about, and build models of animal anatomy of their choice. They use simple materials, crafting supplies, household tools, and/or 3D printing to demonstrate structures of interest, enhancing understanding of the physical principles of animal form and function. Given increased availability of CT data online, students can download, analyze, and 3D print skeletal models of both common and endangered animals. Comparative anatomy students reported that they had increased motivation to study intricate skeletal anatomy simply by manipulating bones in a 3D software assignment. Students in both classes reported enjoying the use of fabrication in learning vertebrate anatomy and this may establish a pattern of lifelong learning.

## Introduction

“What is school for?” asks author and entrepreneur Seth Godin in his education manifesto “Stop Stealing Dreams” (Godin 2012). He argues that society has changed profoundly since the institutionalization of public and higher education and so too should school change. Similarly, advances in how scientists understand animal anatomy as well as developments of sophisticated medical technologies should inspire instructors to think beyond the delivery of textbook-based content. Furthermore, the scholarship of teaching and learning provides evidence for instructors of all fields on how students learn and how to best facilitate lifelong learning (e.g., Berman 2015). The applications of 3D technology in the anatomical sciences are abundant and with the increased accessibility of open-source 3D software (Buser et al. 2020) and affordability of 3D printers, these applications are growing in fields such as ecology and evolution (Walker and Humphries^2019^) and paleontology (Johnson and Carter 2019). Furthermore, researchers are sharing CT scans of animal bones on open access online databases (Table 1) or as supplemental files to publications (Thomas et al. 2016; supplementary material for this article), making 3D files available for use in teaching. By incorporating fabrication into undergraduate anatomy classrooms, instructors can not only inspire students to become innovators and learn both content and skills in a deeper and more engaging way but also do so in an equitable and inclusive way, grounded in the theories and evidence for student learning.

**Table 1:** Digital repositories of 3D vertebrate anatomy for use in teaching with fabrication

Source	Projects of interest to CVA instruction	Description and recommendations	url
Morphosource		NSF-funded repository with tens of thousands of 3D models of biological specimens and more. Free file download with registration and login.	https://www.morphosource.org/
	oVert: Open Exploration of Vertebrate Diversity in 3D	Coordinated effort to scan all vertebrates in museum collections.	Browse > projects > oVert for various links
	*Tiktaalik rosae* complete and disarticulated skull	From Lemberg et al. 2021	https://www.morphosource.org/projects/0000C1213
Sketchfab		General 3D viewer website where users publish 3D files of any genre for viewing, download, and/or purchase.	https://sketchfab.com/
	Witmer Lab	Dinosaurs and many bird species; rhinoceros, *Iguana*, human, bobcat, boa, polar bear, and more.	https://sketchfab.com/witmerlab See also: https://people.ohio.edu/witmerl/projects.htm
	Blackburn Lab	Part of the oVert project; includes hundreds of anatomical models, with an emphasis on herpetology. Many are annotated (e.g., “Wheel of Homology”) as valuable teaching resources. Downloads available at Morphosource.	https://sketchfab.com/ufherps
	Holliday Lab	Excellent teaching resources, including alligator skull and cranial nerves with annotations (from Lessner and Holliday 2020).	https://sketchfab.com/holliday
	University of Dundee Museum Collections	Many downloadable files of charismatic megafauna including hippopotamus, lion, gorilla, elephant, and dolphin.	https://sketchfab.com/uod_museums/models
Digimorph		One of the oldest NSF-funded repositories for CT scans of animal anatomy, many of which have downloadable .stl files for 3D printing.	http://digimorph.org/resources/STLs.phtml
MorphoMuseuM		“M3” is the partner repository for 3D files related to publications in the journal Palaeovertebrata (Lebrun and Orliac 2016).	https://morphomuseum.com/Pages/home
Thingiverse		General repository for 3D files of all genres; free to download. Beware of inaccuracies in uploads by non-experts.	https://www.thingiverse.com/
	Dr. Ian Browne	Includes domestic cat skull, skulls of North American species (e.g., otter, turkey, porcupine, opossum, mink, raccoon)	https://www.thingiverse.com/doccopemys/designs
Thomas et al. 2016		Chrondrocranium of spiny dogfish *Squalus acanthias* and skeleton of cane toad *Rhinella marina*, common species of focus in CVA. Files available as supplemental material.	https://onlinelibrary.wiley.com/doi/full/10.1111/joa.12484

Fabrication, or “making”, in the classroom can not only enhance familiar skills but also improve the process of invention (Blikstein and Krannich 2013). Fabrication can be in the form of creating a digital model in 3D software, 3D printing a physical model through additive manufacturing, or in its most basic form, reminiscent of woodshop or arts and crafts classes. The goal here is to use fabrication as a learning tool, not as an isolated activity divorced from scholarship. A recurring theme in the literature on teaching and learning anatomy is that multimodal approaches have the most successful learning outcomes (Sugand et al. 2010; Kerby et al. 2011; Berman 2015; Estai and Bunt 2016; Wammes et al. 2019). Importantly, making is a way to learn science in a less formal environment and can foster a sense of belonging for historically minoritized students in STEM (Harvin 2015).

This paper provides examples of how to incorporate fabrication into undergraduate comparative vertebrate anatomy (CVA) and introductory biomechanics courses, with an emphasis on creating an inclusive learning environment and ensuring accessibility of resources. A brief overview of theories of motivation related to student learning is given and there is a discussion of important considerations to make these activities as accessible and inclusive as possible. Inspiration and resources are offered for establishing a makerspace in an anatomy lab and leveraging community partners. Finally, two specific cases are detailed where fabrication and the use of 3D digital models are used to augment student learning of vertebrate anatomy at a small liberal arts college.

## Equity-minded motivation for implementing fabrication into vertebrate anatomy courses

Why change the teaching methods for vertebrate anatomy? Changes to pedagogy can not only increase student performance in STEM (Freeman et al. 2014), but can also promote a more inclusive learning environment (Tanner 2013). For the purpose of this paper, “inclusive” will refer to a student's sense of belonging in the classroom. The term “equity” will be used to refer to every student having what they need to successfully learn. Examples are given where instructors can facilitate accessibility of the software, content, and intrinsic motivation to every learner.

“Successful learning” in anatomy is usually measured by grades, and instructors often note a bimodal distribution (e.g., Venail et al. 2010), especially in more introductory courses, with some students performing exceedingly well on exams and other students underperforming on traditional assessments. Recent work has shown that this perception is not always true (Patitsas et al. 2019). Even if there is a normal distribution of grades, it is worth catching the underperforming outliers, those students who may have unseen barriers to learning, especially from traditional methods of lectures and exams.

Uneven patterns of grade distribution, formerly known as the achievement gap, are now referred to as the opportunity gap, reflecting the inequities in the American K-12 education system and access to technology (Goode 2010). At the college level, strategies to fix this opportunity gap may approach student learning from a deficit mindset, blaming the student or “the system” for underperformance, and offering remedial courses or extra tutoring to help underperforming students “catch up” to their peers (Redeaux 2011; Gorski 2019). This approach can burden the students that instructors want to help even more by giving them more tasks and labeling them as being behind before they have begun. Indeed, the underperforming students may bring other social and personal stressors to the classroom such as racialized stress from stereotyped threat (Steele 2011) or working a full-time job to pay for college (Clement 2016). These stressors affect a student's cognitive load and decrease their capacity for learning.

Instead, educators can approach the opportunity gap with an equity mindset, ensuring every student has what they need to learn, which can include access to technology, the motivation to learn, a sense of belonging, and much more. An inclusive learning environment is designed to value every student's inherent talents and experiences. When pedagogical choices foster a sense of belonging for students, this classroom climate leads to the development of students’ sense of awareness and empathy (Dewsbury and Brame 2019) and there is a wide range of small changes in course design that can lead to impactful changes from the student perspective. Minimally, providing low-stakes assignments (i.e., those with a lower points value) early in a course, also called scaffolding, affords students the opportunity to receive constructive feedback on their learning progress and allows instructors to identify gaps in knowledge and/or skills before an important graded exam. Adding structure to biology courses by using such methods as adding preparatory homework or in-class activities helps all students learn more, but it has been shown to disproportionately help Black students and first-generation college students more (Eddy and Hogan 2014).

## Increasing student motivation helps all students become successful learners

External grades are not only the measurement of “successful learning”, but they are also traditionally the prime factor for motivating students to want to learn. Active learning is considered to be an effective motivator for many students (Armbruster et al. 2009), and case studies are given below on activities that could be used with vertebrate anatomy and biomechanics students. Some students lack “buy-in” to these tasks that differ from the traditional lecture method (Cavanagh et al. 2016; Shekhar et al. 2020). If instructors consider intrinsic motivators for student learning, the results may not only improve in that particular course but will also likely extend beyond the course, creating life-long learners. The literature on student motivation for learning abounds, and several concepts relate to the use of fabrication in vertebrate anatomy courses.

The expectancy-value theory (EVT) of motivation originates from the field of psychology, is used by economists, and also contributes to our understanding of student learning. The theory asserts that the intrinsic motivation to learn is a function of the *value* a learner perceives a particular task to have in their own lives and a learner's *expectation* in their ability to achieve that task successfully (See Wigfield and Cambria 2010 for a review). Students must trust that whatever an instructor asks them to do will actually help them by giving some kind of benefit in their lives outside of the classroom (Hulleman and Harackiewicz 2009). For example, a vertebrate anatomy instructor incorporating fabrication can frequently remind students about the value of the skills they are practicing, skills that might be especially appreciated in a future career in medicine, e.g., problem-solving, collaborating with peers, and using 3D software. Emphasizing mastery and learning over grades can also increase student motivation (Meece et al. 2006). As students practice these competencies, instructors can remind them to update their resumes and vitae with the specific software and soft skills.

EVT also states that it is important that students believe that they will actually be able to accomplish what is expected. If students expect to fail, especially on summative, high-stakes exams, then they are less motivated to try and less likely to persist (England et al. 2017). Instructors can coach new learners on the value of failure, but if there are no safe places to fail and learn (i.e., low points formative assessments), then student motivation decreases. Scaffolding provides a foundation to enable students to reach the heights instructors set for them (e.g., Maybin et al. 1992; Offerdahl et al. 2017). Below examples are given of formative assessments that introduce software, materials, and skills for fabrication in the undergraduate anatomy classroom.

There is also opportunity to increase student motivation for learning by giving them a choice in what they learn (Patall et al. 2010) and this is rooted in self-determination theory (See Brooks and Young 2011 for a review). In content-heavy biology courses, this can feel like a tradeoff that instructors are not willing to make. By focusing on training students in the core concepts of a discipline (Petersen et al. 2020), instructors can open up more space for students to choose to learn the specific examples that interest them most. This can be in the form of smaller introductory activities like an icebreaker where students bring a story about their favorite anatomical part on the first day of class or in the form of a self-directed semester-long research and fabrication project, for which more detailed explanation is given in a case study below.

## Lessons for implementing fabrication into comparative vertebrate anatomy courses

### Anatomy lab-turned makerspace

There is evidence that learning science in informal environments and learning specifically in makerspaces promotes equity across diverse cultures and increases motivation to study science (Fenichel and Schwingruber 2010). There is a rich and growing body of literature on “MakerEd” (see Halverson and Peppler 2018 for a review), much of it rooted in Papert's theory of constructionism and the significance of learning by making (Harel and Papert 1991). The social movement of making, the Maker Movement, has been popularized by Dale Dougherty who argues that all humans are makers and that the do-it-yourself (DIY) movement reveals that anyone can learn to do anything (Dougherty 2011, 2012). Proponents of MakerEd argue that in order to foster the next generation of scientists as innovators, educators must break the mold of traditional formal education and allow more room for students to learn through creative processes (e.g., Honey and Kanter 2013).

A makerspace is not defined by the machines; rather it is the spirit of the community, or a sense of belonging, of the learners that use the tools—both traditional and high tech—to create new knowledge and the making itself that defines a makerspace and maker culture (Dougherty et al. 2013). Many universities boast a high-tech centralized makerspace or FabLab (fabrication lab), filled with computers with fast processors, powerful graphics cards, lightning-fast internet, and all of the attractive machines for fabrication such as 3D printers, CNC (computer numerical control) mills and routers, laser cutters, and more. While the costs of 3D technology have fallen in the last decade, so too have academic and departmental budgets. Individual instructors can still develop a makerspace that caters to their students’ needs while staying within the confines of their budget by using donated tools and repurposed materials (Fleming 2015). I posit that a low-tech makerspace may be less overwhelming and more inclusive to students who are not accustomed to this type of learning by making and have not yet had access to high-tech tools.

Establishing a makerspace in a vertebrate anatomy lab can spark student creativity using inspiration from vertebrate animals. Anatomy courses taught in the makerspace emphasize material properties and structure-function correlations as students choose materials to represent morphological features. For example, bubble wrap can mimic the insulating properties of an adipose tissue layer. Wooden dowels, rulers, or popsicle sticks can act as skeletal levers as students learn the mathematical principles governing the position of a fulcrum and its affect on output force. Synthetic muscles can be created from nylon monofilament (fishing line) that is wound into a spiral using a power drill and actuated by temperature (Haines et al. 2014). Zip ties, duct tape, and other household items are handy in holding everything together. This inexpensive approach can be helpful in getting students out of the textbook–lecture comfort zone and accustomed to the concept of play in learning (e.g., Wohlwend and Peppler 2015), especially on the introductory level. Starting with familiar materials may reduce the barrier to entry for many students who have no previous experience with fabrication or self-directed learning, increasing their expectancy in the ability to learn animal anatomy with recognizable supplies and fostering an inclusive atmosphere where everyone feels like they belong. Along with the low-tech inclusive making activities in the anatomy classroom, I offer my experience with introducing students to the higher-tech options for fabrication, namely 3D modeling and 3D printing.

### Scaffolding and accessibility of 3D software

It may come as a surprise to instructors when students who belong to the generation that is referred to as digital natives state, “I'm not good with technology.” An instructor's response might be, “Yet!” It is true that no one is inherently skilled with software that they have never seen; the same is true for anatomical jargon. Scaffolding of activities allows students to fail early and safely in low-stakes assignments, building confidence and expertise (Maybin et al. 1992), and is important in applying the expectancy-value theory of motivation to student learning.

**Fig. 1:**
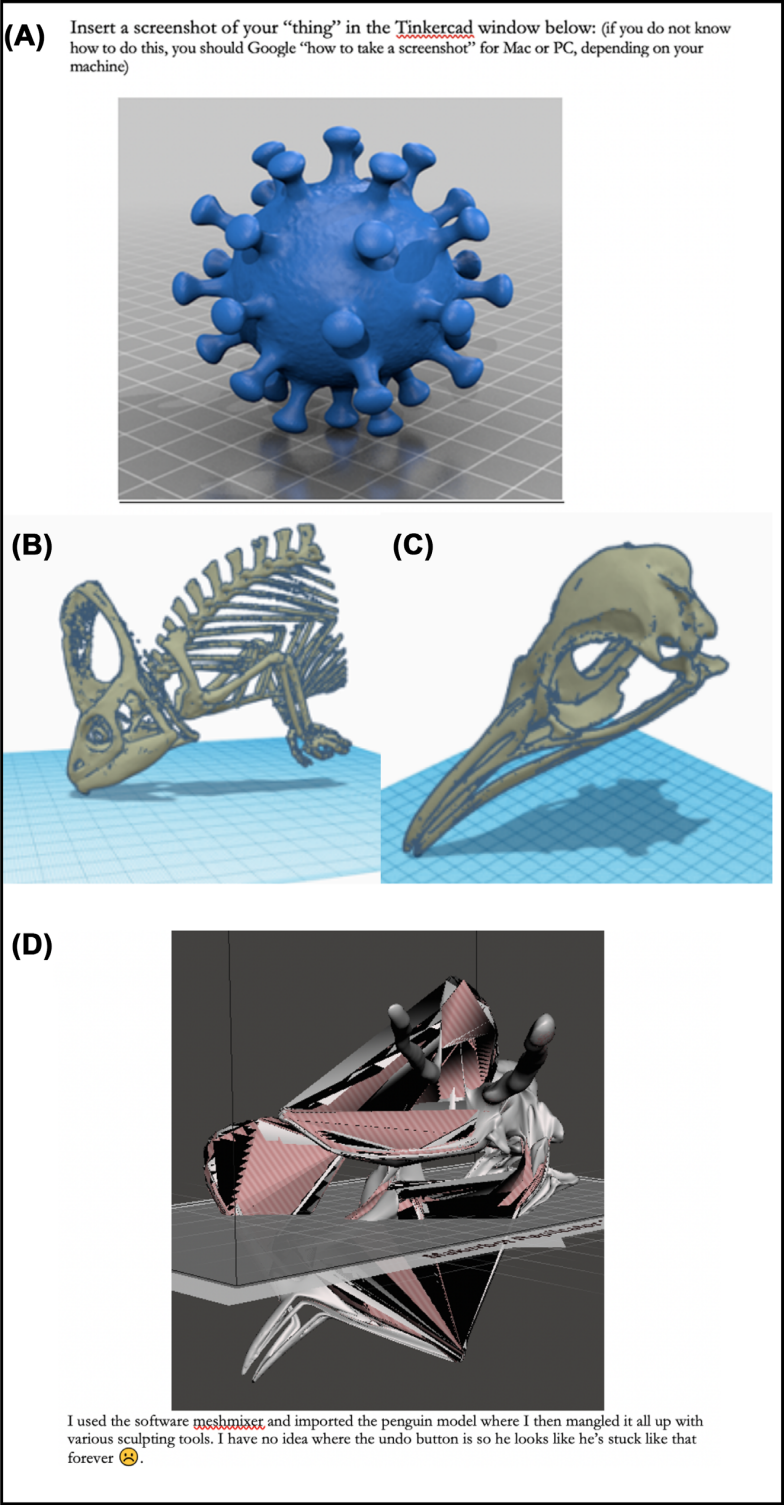
Examples of student work in introductory assignments. These low-stakes formative assessments introduce students to software to reduce the cognitive load when completing work that is important to learning anatomy. **(A)**: Instructions to students and a screenshot of a coronavirus surface file obtained from Thingiverse and imported into the Tinkercad browser-based software. **(B):** Screenshot of a veiled chameleon (*Chamaeleo calyptratus*) model obtained from Digimorph and imported into Tinkercad. **(C)**: Screenshot of an emperor penguin (*Apetenodytes forsteri*) model obtained from Digimorph and imported into Tinkercad. **(D)**: Screenshot of the same penguin model imported into Meshmixer software and modified by student exploring the sculpt tools. Student comments included to show that formative assessments should be less formal and more exploratory.

An assignment introducing anatomy students to 3D software should not only include the basics of how to obtain and maneuver 3D files in general but also familiarize students with the digital repositories where animal anatomy files can be obtained (Table 1; Fig. 1). For instance, a lesson could include browsing the popular Thingiverse website, a digital repository for 3D files (usually in .stl or .obj format), and ask students to download an object file of their choice to practice importing it into 3D software (Fig. 1A). Students typically need to learn the difference between unsuccessfully double-clicking to open a downloaded surface file versus importing the file using the 3D software. By practicing with non-anatomical 3D models, students can learn the software prior to using it for learning anatomy, reducing the cognitive burden during anatomical assessments. The introductory assignment should also include exploration of 3D animal anatomy, with the singular goal of learning the skill of obtaining and manipulating a file, not learning the names of the anatomical structures. Further introductory steps can prompt students to explore the tools within the software (examples below), and this can be catered to an instructor's goals for future assessments.

There are now many options for free 3D software, making this technology increasingly accessible, but not every student has their own computer. This can create an equity problem with an assignment that includes 3D digital models. One option to make 3D software more accessible to a broad range of students is to elicit the help of the university information technology (IT) department. Instructors can request for IT to install the relevant open-source software onto the machines in a computer lab and have the class meet in the computer lab for introductions to CT scan repositories. In this way, students will first learn the software with the instructor facilitating. Another option is to consider browser-based alternatives like Tinkercad, so that even if a student is borrowing their parent's work laptop or a university computer in the library, they can save their files in the cloud without the need of a personal device.

### Community partnerships for 3D scanning and printing

Vertebrate anatomy instructors are presumed to be experts in their field, able to deliver the concepts of animal form and function. They are not expected to be fabrication experts or even hobbyists, so how should a novice consider incorporating 3D technology into a vertebrate anatomy course? I argue that it is beneficial for students to see their instructor as a human who may be a beginner to fabrication because this can demonstrate to the students how to be a humble yet confident learner. However, this may lead to stalls in fabrication if an anatomy instructor purchases a single 3D printer for example, and that printer stops working before final projects are due. As such, it is recommended that instructors have a back-up plan. Community partnerships not only serve to contribute to student projects, but also show students how essential collaboration is to meeting a goal successfully.

Community makerspaces can be found in most cities, but public libraries increasingly provide 3D printers for public use (Hoy 2013; Moorefield-Lang 2014). There is also a vast, friendly maker community online, filled with hobbyists and academics who are happy to troubleshoot and contribute to the next generation of scientists and makers (e.g., Tachibana 2019).

Instructors can also leverage local businesses that specialize in 3D technology and engage with local non-profits working to bring technology to the community. By happenstance, I attended a local event in a series of educational speakers hosted by the Carroll [County, MD] Tech Council. It was here that I met the CEO of a local 3D scanning business, Direct Dimensions, Inc., a company that specializes in large-scale projects like movie sets and scanning the National Cathedral in Washington, DC after the 2011 earthquake to help architects design preservation solutions. The company was happy to accommodate my small-scale request to create digital models of domestic cat bones for teaching (Supplementary Material), and they did so pro-bono.

In these examples and in many other collaborative approaches, anatomy instructors can reduce their own cognitive loads in learning 3D technology and create lasting relationships among the academic, public, and private sectors in the community.

## Lessons from the vertebrate-anatomy-classroom-turned-makerspace: Two case studies

### Case Study 1: Introductory biomechanics semester-long project

Semester-long research projects are successful tools for engaging students in vertebrate anatomy as an active field of inquiry and research (e.g., Ghedotti et al. 2005) instead of simply being an endeavor in memorizing the longstanding names of anatomical parts and their functions. Incorporation of staged components for a longer project allows for formative feedback from the instructor to help students meet expectations for the final project (Lachman 2015). In the example presented here, students in an introductory biomechanics course investigate, write about, and build physical models of animal anatomy of their choice, keeping in mind that self-determined learning can increase student motivation (Patall et al. 2010; Brooks and Young 2011). This introductory course is capped at 25 students and meets 3 day a week for 1 h each. It does not have a separate lab component but is taught in the anatomy-lab-turned-makerspace so that students have access to materials and tools for constructing the physical models.

Each student chooses a structure–function relationship in animal anatomy that is of interest to them. Students are exposed to examples from previous student work and from structured readings and discussions of scholarly literature, but ultimately the choice of topic to research for the project is up to each individual student, as long as they are motivated to learn about it. The project gives students practice with researching scholarly literature, writing a research paper, and presenting their findings to the class. Importantly, each student builds a physical or digital model of their chosen anatomical structure, using the simple materials, crafting supplies, household tools and/or 3D printing. They have the option to use the model to demonstrate structures of interest, enhancing understanding of the physical principles of animal form and function, or to use those models to test hypotheses on form and function. Many students take advantage of the availability of CT data online, downloading, analyzing, and 3D printing skeletal models of common, endangered, and extinct animals, including charismatic megafauna that are appealing to introductory students.

The importance of scaffolding cannot be overemphasized, and this is what students report appreciating the most. Students have frequent check-ins with the instructor by allotting time in class for work on the physical model aspect of their independent projects. “Figure it out Fridays” give students time during class in the makerspace anatomy lab to examine specimens of interest that are already in the lab's collection, teach themselves a new skill such as introductory robotics, and to tinker on their physical models with the instructor's and teaching assistants’ immediate feedback. These in-class working sessions begin early in the semester, and if a student chooses a charismatic megafauna, e.g., a cheetah or great white shark, they are encouraged to examine a reasonable representative within the anatomy lab such as a domestic cat or the spiny dogfish, especially if they are unsure where to start with the physical model. Students can request additional lab time for project work in coordination with the instructor and departmental lab manager, but with the knowledge that immediate feedback on project work is only available during the in-class working sessions and associated written reflections. Students submit written status reports for each in-class working session so that they can reflect on their own progress and so the instructor can make suggestions for next steps. The prompt for these formative, low stakes assignments is, “How did you use the hour on Friday? What did you try? What worked and what didn't? What do you plan to do next time? This should be written as a reflection and will give you the chance to receive feedback on your project's progress”. When students reflect on their own learning, they engage in metacognition, examining their comprehension and progress, and practicing the skill of being a self-directed learner (Silver 2013), increasing intrinsic motivation to learn. Formative assessment in a making classroom can allow for culturally responsive teaching (Hadad et al. 2020), meeting each student exactly where they are, with or without prior knowledge or skills. If students begin with an informed idea and hit the ground running with construction of their physical model, they are pushed to test hypotheses about animal form and function using their model and to analyze results using statistics. If a student is less confident and intimidated by the work, then the pedagogical focus is to help them gain independence with scholarly research and understand the primary sources to inform the building of an accurate replica of animal anatomy.

To set the tone for the type of independent work that students will do in the research project, the class begins on the first day with a model-building and written reflection activity. This has ranged from, “Build a physical model of your favorite concept from introductory biology”, to “construct a no-sew mask from a T-shirt, modified to fit your face” during the COVID-19 pandemic. Students receive emailed prompts for thinking about a topic (and, in the case virtual learning during the pandemic, coordination for access to materials) prior to the beginning of the semester. To positively frame students’ perceptions of making activities in an anatomy lab, they are asked to imagine their future career where they will not be expected to take standardized tests, but will need to think their way through solving a problem. Providing context behind pedagogical decisions helps students see the value in this type of work (Shekhar et al. 2020).

Students continue being familiarized with independent and collaborative fabrication work through a hands-on activity associated with each content area. Instructors can adapt activities based on content choices or time allotted, and can modify assessments originally designed for younger learners to meet the learning objectives for various college-level biology courses. In this introductory biomechanics course, there are six content units, each with one lecture, one scholarly article discussion, and at least one lab-like activity, with project-work and Figure-it-out Fridays interspersed throughout. Students generate stress–strain curves using gummy worms (modified from Williamson 2013) during a unit on size, shape, and stiffness. In a unit titled “Life in Moving Fluids” (borrowed from Vogel 2020), students use Karo syrup in differing concentrations and drop spheres of different densities and obtain velocities to calculate Reynolds numbers for each solution (modified from Rucker 2010). Even a fabrication activity designed for young children, such as making a model of mammalian respiratory organs using a drinking straw to represent the trachea, a small balloon as a lung and a large balloon as the diaphragm contained in a pleural cavity represented by a plastic bottle (Zak 2020) can insert a fun and quick lesson to deepen students’ learning of Boyle's law and the inverse relationship of pressure and volume of fluids. At the same time, students read and discuss relevant scholarly literature for each unit, exposing them to ways that biologists use physical models to study animal structure and function (e.g., Paig-Tran et al. 2011). In these ways, students gain many structured opportunities to practice thinking through problems through kinesthetic learning and fabrication to scaffold the innovative independent fabrication work in their research projects.

**Fig. 2:**
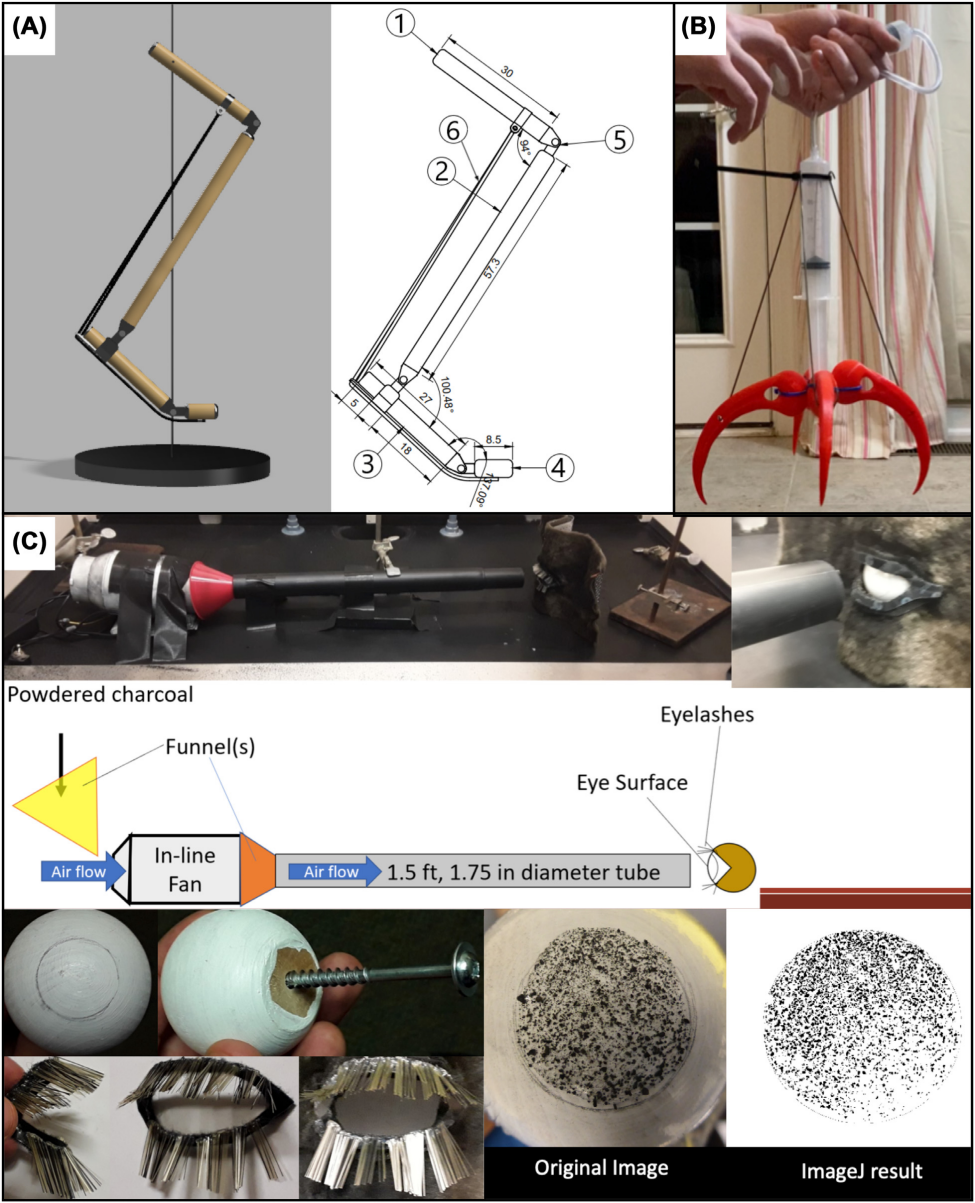
Examples of semester-long research projects from an introductory biomechanics course. **(A):** Digital model of a kangaroo hindlimb created in Fusion 360 software. **(B):** Physical model of a raptor hindlimb. A hydraulic pump was constructed using a syringe and tubing to actuate 3D printed talons. **(C):** Experimental setup testing the effect of “camel eyelash” length on particle dispersal. *Student work was provided by S. Minutelli (A), B. Hulse (B), and G. Abbas (C) and is published with their permissions*.

Enthusiasm is contagious as students show off their diverse and impressive final projects (Fig. 2). A student athlete built a novel football helmet inspired by woodpecker skull anatomy and the shock-absorbing features of its morphology (e.g., Jung et al. 2019). Kangaroos are a popular structure–function choice and one student taught themselves to use Fusion 360 software to design a digital model of the hindlimb (Fig. 2A). A student with a vested interest in raptors investigated the tendon locking mechanism of the hindlimbs (Ward et al. 2002), designed talons from scratch in 3D software, and constructed a hydraulic pump to actuate the 3D prints (Fig. 2B).

Another student was inspired by camel eyelash morphology (Amador et al. 2015) and tested different lengths of eyelashes on particle dispersal with an elaborately designed experimental setup, and statistical analyses of results obtained from ImageJ (Fig. 2C). Anonymous end-of-semester course evaluations demonstrated students’ appreciation of being challenged to think and learn in different ways that could apply to their lives.

It is worth noting that, the semester after taking the class, a student from the introductory biomechanics course won the College's entrepreneurship challenge which comes with a $10,000 prize. Because the emphasis is on animal-inspired innovations and examples of products inspired by nature are used frequently, this fosters student creativity in solving bigger problems. While the winning student did not use a design from the course, she told me that she would not have even considered entering the contest prior to taking the biomechanics course and practicing the iterative fabrication process.

The design of the semester-long research project on animal structure and function may not scale to large university classrooms, given the individualized nature of the work with the physical model construction. However, instructors could consider adapting the research project for pairs or groups to allow students to practice the skill of collaboration. Additionally, the smaller-stakes group activities that are associated with each content area could scale, especially with the help of teaching assistants.

This approach to teaching animal biomechanics has benefits for instructors as well. With student-driven courses, there is less content for the instructor to prepare; the tradeoff is time spent giving valuable formative feedback. When students steer their own learning, this eases the decision fatigue burden on the instructor, with choices on “which content to cover”. At the same time, instructors will likely learn more about animals outside of their area of expertise and this supports a shared discovery-based model of learning.

### Case Study 2: 3D long bone anatomy learning experiment

#### Background

Comparative vertebrate anatomy (CVA) is one of the oldest branches of human inquiry in the sciences. Learning the tedious structural names rooted in Latin can be overwhelming, especially to sophomores who choose to take the course immediately after an introductory sequence. Here, an example is offered of an assessment of student learning in the laboratory portion of this course at a small liberal arts college. CVA fulfills requirements for the biology major, is of interest to pre-medical students, and is composed of both lecture and lab components. The typical structure of a lab period involves students working through a list of terms that they are responsible to know for one of three lab practical exams throughout the semester. The learning assessment was conducted during the first-third of the lab component, which focuses on structures of the vertebrate skeleton. The rest of the semester involves preserved specimen dissections. Students have access to physical models of the bones and are allowed to borrow the bones from the lab for studying.

I tested whether a 3D homework assignment on long bones increased performance on exam questions. The motivation behind this learning experiment was witnessing several students fail the first lab practical exam, and this being their “wake up call” to take the work seriously and change studying habits. The failure of a summative exam is demotivating and not helpful in ultimately learning the skeletal material. Furthermore, the material builds on itself, as the class moves to skeletal muscle dissections afterwards and lack of knowledge of skeletal structures will hinder learning of muscular origins and insertions. Thus, I tested if students could enhance learning of the skeletal structures through a homework assignment in which they “paint” the detailed structures onto virtual bones using the freely available Meshmixer (Autodesk) 3D software.

#### Methods

Accessibility of 3D software and reduction of cognitive load began with an introductory activity, catered to prepare students for the long bone experimental learning assignment. Previous work with medical students emphasizes the importance of training anatomy students in 3D software (Silén et al. 2008). During the first week of the course, the class met in a campus computer lab where Meshmixer was already installed on the machines. Students worked through an introductory assignment where, in addition to downloading, opening, manipulating, and analyzing CT scans from digital repositories (Table 1), they also practiced using the sculpt and paint vertex tools that they would encounter with the experimental assessment. In this way, the students could gain familiarity with turning, manipulating, and painting the bones in the same 3D digital platform that they would use during the skeletal learning assignment. The goal of the introductory assignment was to facilitate student readiness to work immediately with the anatomical content in the experimental homework without the additional cognitive burden of learning the software.

Students completed the experimental homework assignment prior to the first lab practical exam on skeletal anatomy. Four cat limb bones were laser-scanned by a local company specializing in 3D imaging technology (Direct Dimensions, Inc., Owings Mills, MD) and students were provided with the .stl files (Supplementary Material). Each student was randomly assigned two 3D scanned long bones, any combination of a left and right femur and/or a left and right humerus. As such, a student may have worked with contralateral scans of the same bone or ipsilateral scans of two different bones and there were six categories of assignments (left and right humerus, left humerus and left femur, left humerus and right femur, right humerus and right femur, right humerus and left femur, and left and right femur). Because they use 3D software, students can orient themselves to see what is cranial, caudal, medial, lateral, helping to distinguish left from right, a benefit that other anatomy instructors have noted in using such software for learning (Silén et al. 2008; Venail et al. 2010).

**Fig. 3:**
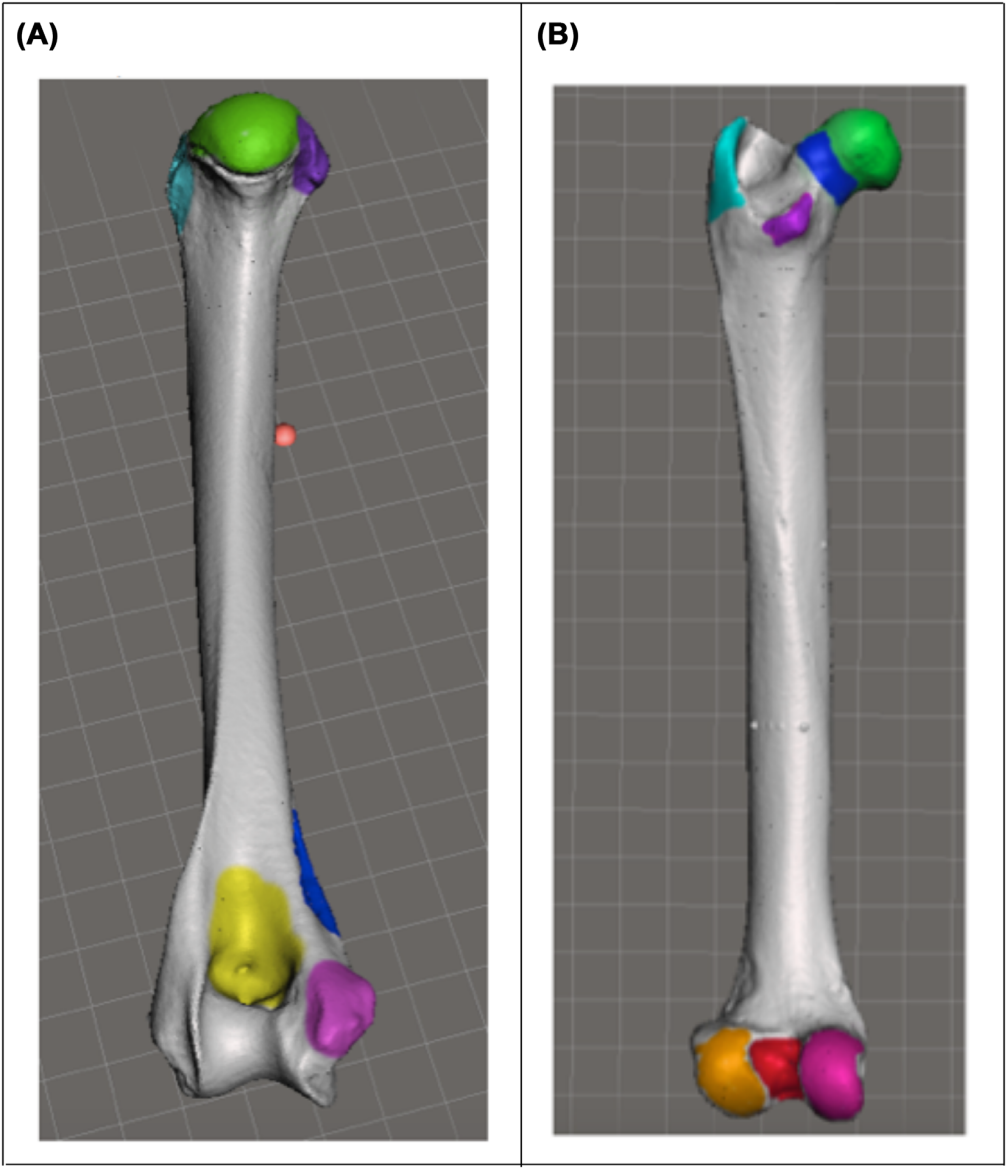
Examples of student work in the CVA 3D long bone learning assignment. Students added color to skeletal structures *Felis* bones by “painting” in Meshmixer software. **(A)**: screenshot of a caudal view of the left humerus with color added to the the head, lesser and greater tubercles, olecranon fossa, supracondylar foramen, and medial epicondyle. **(B)**: screenshot of left femur, caudal view with color added to the head, neck, greater and lesser trochanters, lateral and medial condyles, and the intercondylar fossa.

Students were given the same list of terms for the assignment that they had been given in that week's lab meeting with physical bones. Students then used Meshmixer to identify and shade or add color to the detailed structures on their assigned bones, based on the list of structures that they were to know for the lab practical exam. Students had the option to use different colors for each structure or to use the same color in separate images, as long as each structure was clearly labeled. Students submitted labeled screenshots of their work in the 3D software and were given feedback on accuracy (Fig. 3). This was repeated in three consecutive years (2016–2018) with a total of 48 students. Both qualitative and quantitative results were collected on student learning. The McDaniel College Institutional Review Board approved all methods. Each student participant signed an informed consent form.

Students completed a survey with questions regarding their attitudes towards the assignment and whether they considered it to be helpful to their learning. The majority of the survey included questions on a five-point Likert scale, but open-ended questions were also included to gain insight into students’ candid impressions. Importantly, the survey asked if students used their own computer to complete the assignment, which would require downloading the Meshmixer software onto their personal machine, or if they returned to the computer lab to complete the assignment on a campus device with the software already installed.

Lab practical exam questions pertaining to the humerus and femur were used to assess student learning, with questions on the tibia serving as a control. Exam questions asked students to provide the name of the physical bone, the side of the body, and a specific feature labeled on each bone that was both on the terms sheet and the 3D homework assignment. Students were not provided with a word bank, requiring them to recall the anatomical names for the bones and structures.

#### Results and discussion

**Fig. 4:**
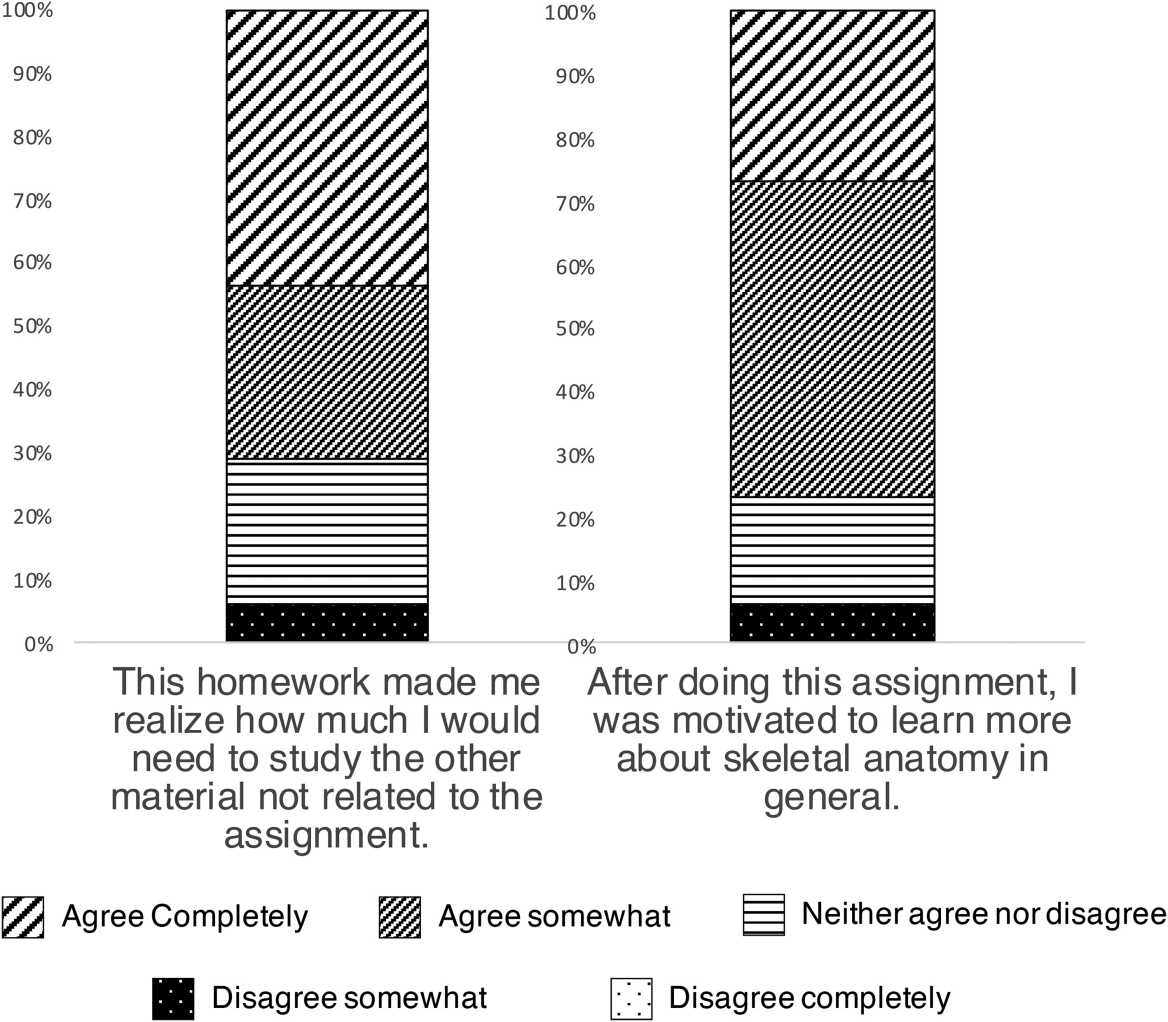
Comparative vertebrate anatomy 3D long bone learning experiment results from student survey showing that 77% of students agreed that the 3D homework assignment helped them realize the amount of studying they would need to do to learn the skeletal anatomy (left). Students also reported that they were motivated (70% agreed) to learn more after completing the assignment (right).

The survey revealed students’ attitudes and experience of manipulating bones in 3D and how the use of 3D software connected to their own learning processes and studying choices (Fig. 4). Comparative anatomy students reported they had increased motivation to study intricate skeletal anatomy simply by manipulating bones in a 3D software assignment (27% agreed completely; 50% agree). They also reported that this assignment helped them realize just how much work needs to go into learning intricate skeletal anatomy (43% agreed completely; 27% agree). In other words, this assignment made them engage with the list of anatomical terms in a way that they wouldnot have otherwise done with simply holding the physical bones in the lab.

There was a trend towards students performing better overall on exam questions on the bones that were part of the 3D homework assignment with 60% correctly identifying the humerus and the correct side of the body and 73% correctly identifying the femur and the correct side of the body. This is compared to the tibia, a control that they had not manipulated in software, which 49% of students identified correctly. There was not a statistically significant difference in accuracy between experimental bones and the control (one-tailed T-Test; *p* = 0.127). This is likely due to one year's class boasting more accuracy on all questions and skewing the bigger differences seen in the other two classes. Overall, the sample size is still too small to make any strong conclusions on the effect of assigned bone and exam accuracy.

Since there was an emphasis on accessibility of 3D software to create an equitable learning opportunity, the survey data on computer usage is of interest. While 83% of students exclusively used personal machines for the 3D learning assignment, 10.6% of students reported using campus computers exclusively (4.3% used both; 2.1% borrowed a classmate's computer). This suggests that at least 10% of students either preferred to avoid or were unable to download the Meshmixer software onto a personal device, allowing them to complete the anatomical assignment without this extra step. This may be most important for those students who may have the most stressors or distractions.

Perhaps the most satisfying outcome of this small pedagogical trial is that of student enjoyment. Over 75% of students reported enjoying the 3D long bone assignment (45% agreed completely; 32% agreed somewhat; 21% neither agreed nor disagreed; 2% disagreed somewhat; 0% disagreed completely). Enjoyment is related to motivation and may be a key to producing lifelong learners (Sibthorp et al. 2011).

## Concluding remarks

Instructors of vertebrate anatomy have choices in how to instill concepts and skills in the next generation of scientists and how to make the field inclusive and accessible to all students. The use of fabrication, or making, as a pedagogical strategy permits flexibility in student choice on what to learn and enhances problem-solving skills. Formative assessments used in a makerspace classroom facilitate culturally responsive teaching where diverse perspectives are celebrated. For these reasons and more, the use of digital fabrication and 3D printing has become increasingly popular in biological education but our understanding of its value on student learning is still in its infancy (Hansen et al. 2020). In the examples here, it is demonstrated that fabrication projects motivate students to learn vertebrate anatomy, infusing a sense of fun into their formal higher education.

## Supplementary Material

icab147_Supplemental_File

## Notes

From the symposium, “Biology Beyond the Classroom: Experiential Learning Through Authentic Research, Design, and Community Engagement,” presented virtually at the annual meeting of the Society for Integrative and Comparative Biology, January 3–7, 2021.
