# Supplementary material for: Implementing Fabrication as a Pedagogical Tool in Vertebrate Anatomy Courses: Motivation, Inclusion, and Lessons
Source: Integr Comp Biol. 2021 Jun 26;61(3):1013–27. doi: 10.1093/icb/icab147 (PMC8490688; doi:10.1093/icb/icab147)
Supplement: icab147_Supplemental_File [file icab147_supplemental_file.zip › icb-2021-0135-File011.pptx]

## Slide 1
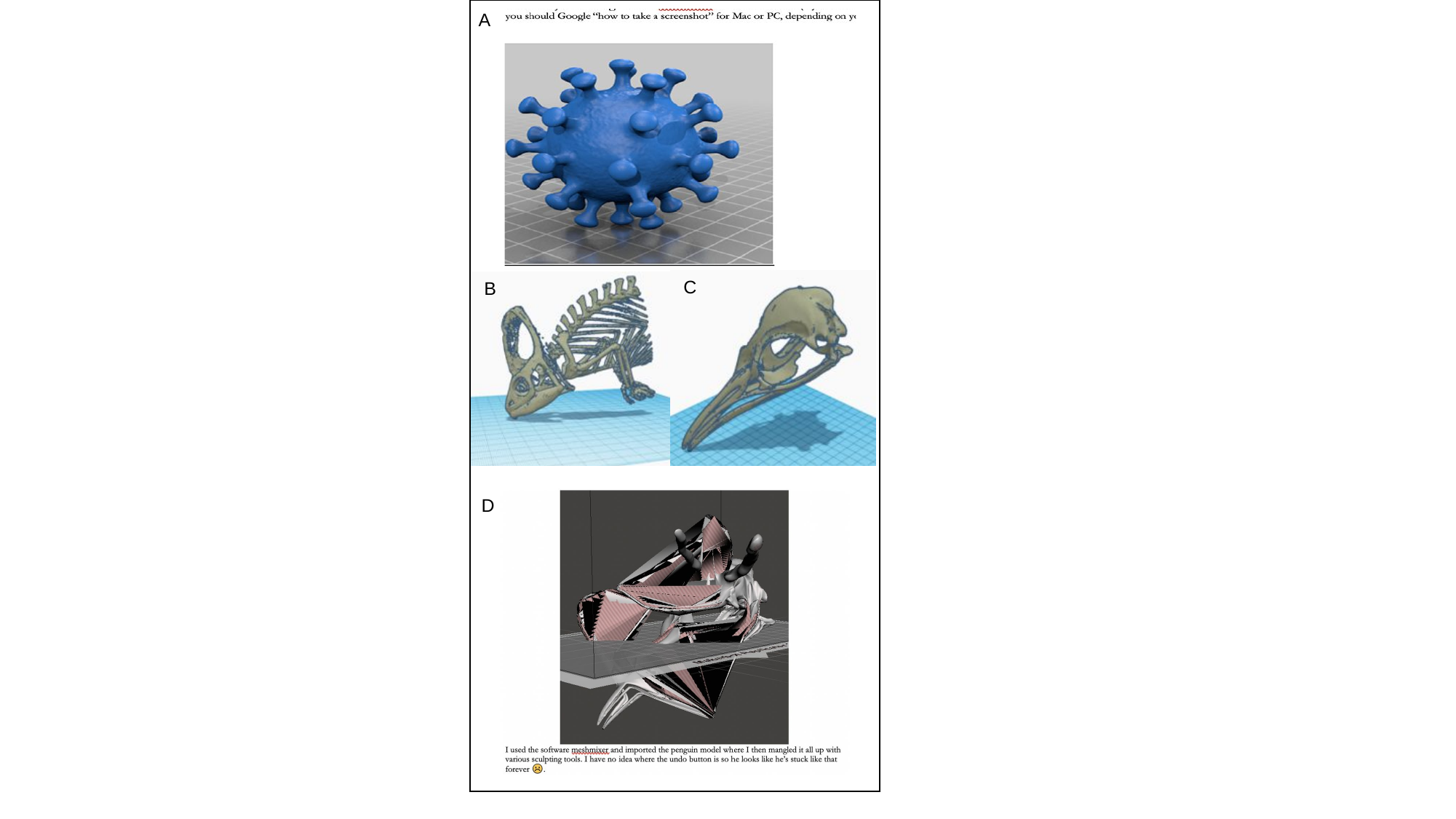

A
C
B
D

## Slide 2
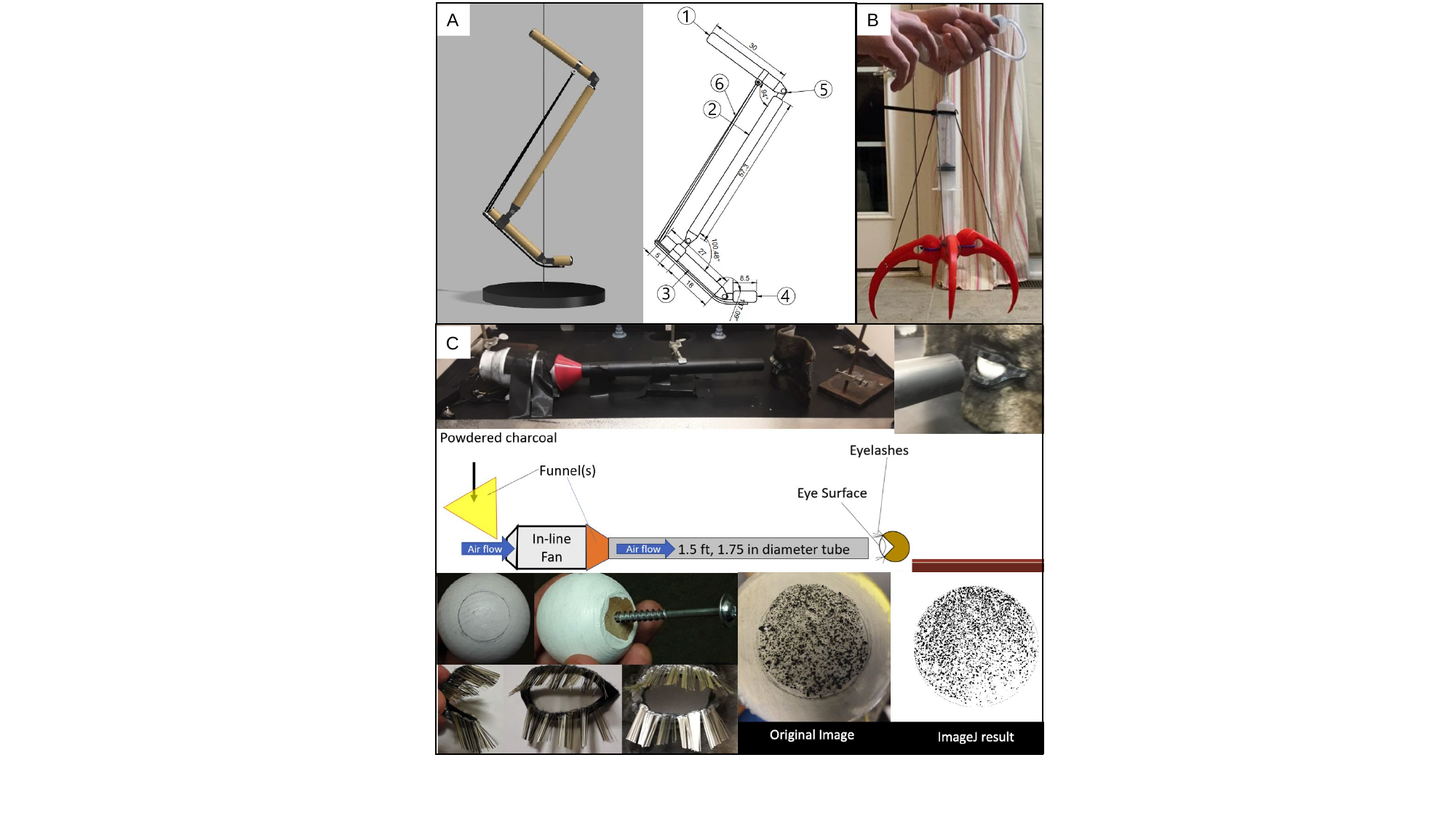

B
A
C

## Slide 3
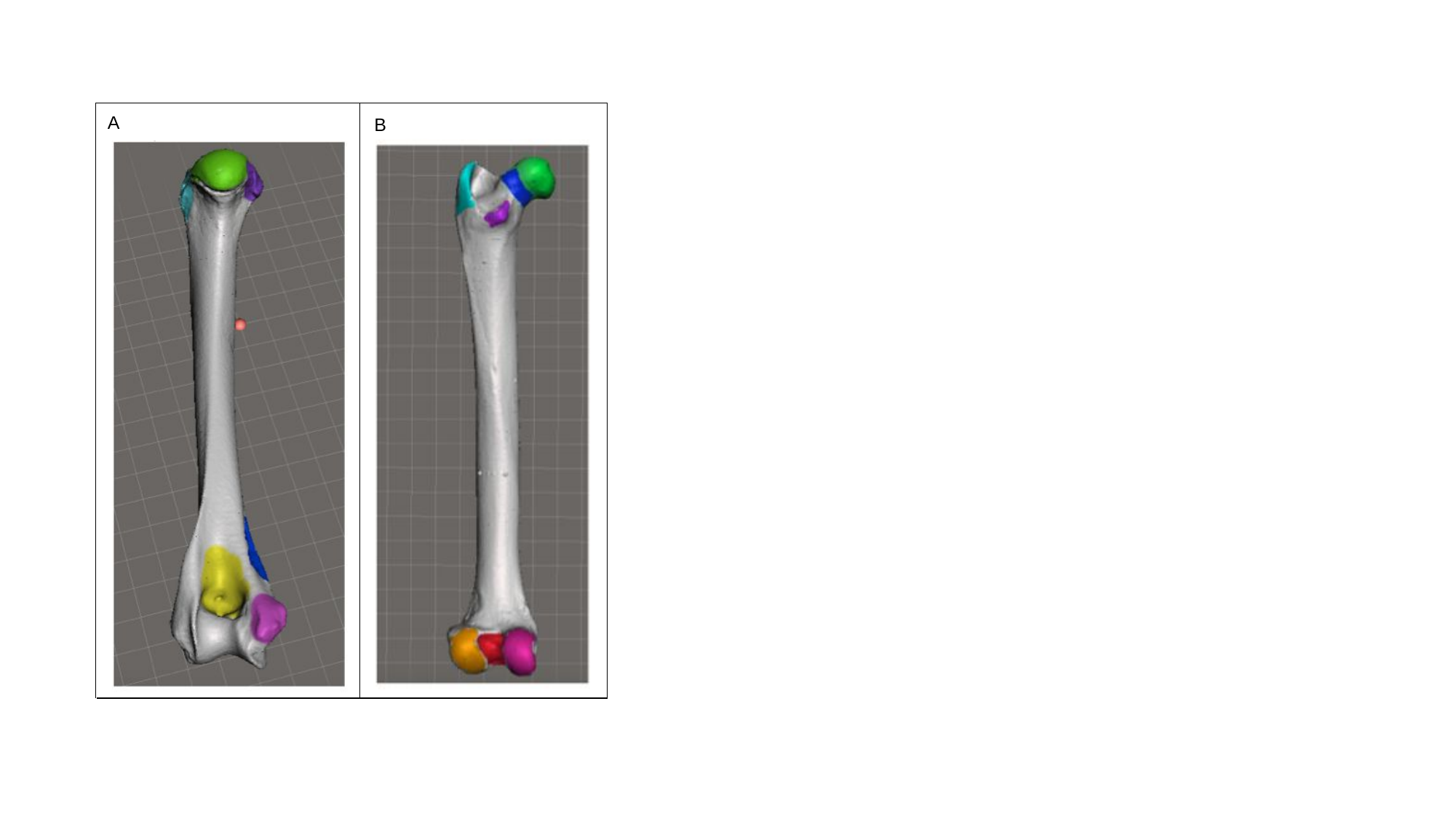

A
B

## Slide 4
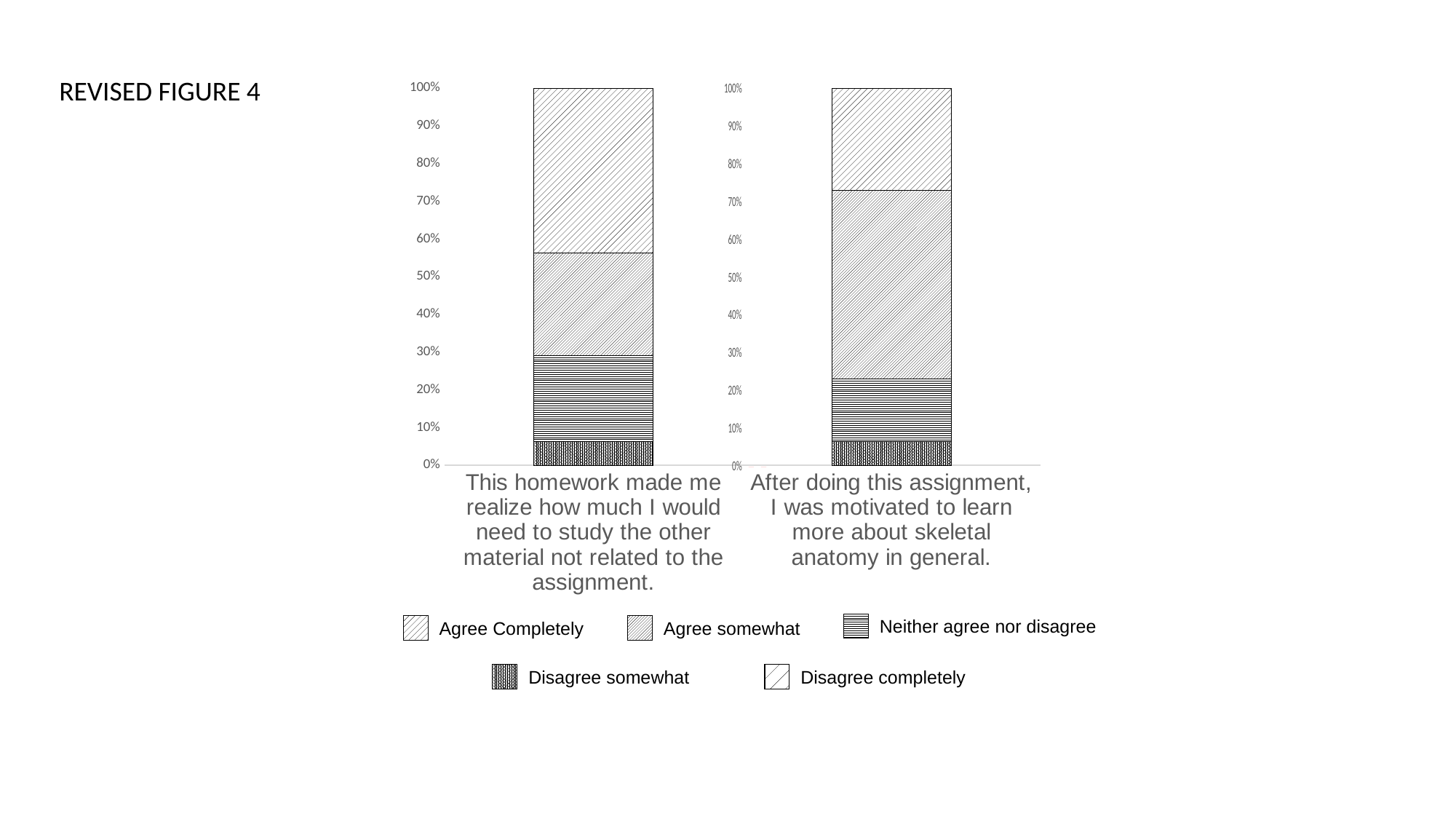

REVISED FIGURE 4
### Chart
| Category | Disagree Completely | Disagree Somewhat | Neither Agree nor Disagree | Agree Somewhat | Agree Completely |
|---|---|---|---|---|---|
| This homework made me realize how much I would need to study the other material not related to the assignment. | 0.0 | 6.25 | 22.916666666666664 | 27.083333333333332 | 43.75 |
| After doing this assignment, I was motivated to learn more about skeletal anatomy in general. | 0.0 | 6.25 | 16.666666666666664 | 50.0 | 27.083333333333332 |
### Chart
| Category | Disagree Completely |
|---|---|
| This homework made me realize how much I would need to study the other material not related to the assignment. | 0.0 |
| After doing this assignment, I was motivated to learn more about skeletal anatomy in general. | 0.0 |Neither agree nor disagree
Agree Completely
Agree somewhat
Disagree somewhat
Disagree completely
